# Generation of Immortal Cell Lines from the Adult Pituitary: Role of cAMP on Differentiation of SOX2-Expressing Progenitor Cells to Mature Gonadotropes

**DOI:** 10.1371/journal.pone.0027799

**Published:** 2011-11-21

**Authors:** Ginah L. Kim, Xiaomei Wang, Jennifer A. Chalmers, David R. Thompson, Sandeep S. Dhillon, Margaret M. Koletar, Denise D. Belsham

**Affiliations:** 1 Department of Physiology, University of Toronto, Toronto, Ontario, Canada; 2 Department of Obstetrics and Gynaecology, University of Toronto, Toronto, Ontario, Canada; 3 Department of Medicine, University of Toronto, Toronto, Ontario, Canada; 4 Division of Cellular and Molecular Biology, Toronto General Hospital Research Institute, University Health Network, Toronto, Ontario, Canada; University of Córdoba, Spain

## Abstract

The pituitary is a complex endocrine tissue composed of a number of unique cell types distinguished by the expression and secretion of specific hormones, which in turn control critical components of overall physiology. The basic function of these cells is understood; however, the molecular events involved in their hormonal regulation are not yet fully defined. While previously established cell lines have provided much insight into these regulatory mechanisms, the availability of representative cell lines from each cell lineage is limited, and currently none are derived from adult pituitary. We have therefore used retroviral transfer of SV40 T-antigen to mass immortalize primary pituitary cell culture from an adult mouse. We have generated 19 mixed cell cultures that contain cells from pituitary cell lineages, as determined by RT-PCR analysis and immunocytochemistry for specific hormones. Some lines expressed markers associated with multipotent adult progenitor cells or transit-amplifying cells, including SOX2, nestin, S100, and SOX9. The progenitor lines were exposed to an adenylate cyclase activator, forskolin, over 7 days and were induced to differentiate to a more mature gonadotrope cell, expressing significant levels of α-subunit, LHβ, and FSHβ mRNAs. Additionally, clonal populations of differentiated gonadotropes were exposed to 30 nM gonadotropin-releasing hormone and responded appropriately with a significant increase in α-subunit and LHβ transcription. Further, exposure of the lines to a pulse paradigm of GnRH, in combination with 17β-estradiol and dexamethasone, significantly increased GnRH receptor mRNA levels. This array of adult-derived pituitary cell models will be valuable for both studies of progenitor cell characteristics and modulation, and the molecular analysis of individual pituitary cell lineages.

## Introduction

The pituitary gland is known as the master gland because of its central role in governing endocrine activities, together with the hypothalamus. The hypothalamus releases trophic factors that act on specific cell types in the pituitary, stimulating or inhibiting the secretion of pituitary hormones. These pituitary hormones act on peripheral target organs to ultimately regulate growth and development, reproduction, metabolism and stress adaptation. While the involvement of the pituitary in whole body physiology is well understood, the molecular mechanisms underlying the endocrine feedback control of hormone synthesis and secretion have not been fully defined. Representative cell lines of all six anterior pituitary cell types have been previously established and have contributed much to our understanding of pituitary cell physiology and the mechanisms by which hypothalamic peptides control pituitary activities [1]. However, each of the cell lines was derived independently and thus difficult to compare, due to species differences, tumour-versus SV40 T-antigen transgenically-derived cells, and variation in the temporal isolation of each line. In addition, these cell lines may not be wholly representative of adult pituitary cells, as many of these lines originated from pituitary tumors at an early developmental stage. Limited cell models from the posterior and intermediate lobes have presently been generated [1].

Previously, our laboratory has used a novel technique to immortalize cell lines from both the embryonic and adult hypothalamus [2], [3]. Currently, over 100 clonal cell lines are available to study the regulation of distinct neuronal cell types found within the heterogeneous mix within the hypothalamus. These lines have been used for detailed analysis of hormonal regulation of neuropeptides and the extensive signal transduction events necessary to differentially control each individual neuronal population [4], [5]. With this in mind, we attempted to immortalize adult-derived primary culture from a 12 week-old female mouse. We report herein the generation and characterization of 19 mixed cell lines, as well as a number of cloned cell models from a single mouse pituitary.

Whether the adult pituitary is capable of producing stem or progenitor cells to replace differentiated cell types lost due to stress, damage, or cell death during the lifespan is under consideration. Recent studies have focused on the characterization of putative pituitary stem cells and progenitor cells that can undergo further differentiation to generate each of the individually defined pituitary cell types [6], [7]. Of particular interest is the identification of individual cell markers that distinguish stem cells from progenitor and more fully differentiated cell types, where SOX2, nestin, S100, and SOX9 were used as identifiers of multipotent adult progenitor cells or transit-amplifying cells [8]. Our clonal cell lines offer a unique opportunity to determine whether any of these progenitor cells could be immortalized with the mixed primary culture. Analysis of the cell lines indicates that some of the lines indeed show characteristics of progenitor pituitary cells.

A number of hormones and neurotransmitters have been demonstrated to drive progenitor cells towards differentiation, particularly with neuronal cell types [9]. Extensive analysis of the putative signals that induce differentiation of progenitor cell types has been undertaken and the list of candidates is growing substantially. Some of these include nerve growth factor [10], brain derived neurotrophic factor [11], pituitary adenylate cyclase-activating peptide [12], [13], among other neurotrophic factors. The downstream effectors of receptor-mediated differentiation include many signal transduction pathways, with defined second messengers and transcription factors. Of interest, cAMP has been implicated as an important second messenger in the differentiation of progenitor cells [12], [14], [15], [16]. While much progress has been made towards understanding neuronal progression and the specific factors that drive differentiation, less is currently understood regarding the mechanisms controlling lineage specification of pituitary progenitor cells. We have therefore used our putative progenitor lines to determine if chemical modification can drive the progenitors to a more differentiated phenotype.

Taken together, we have immortalized a wide array of pituitary cell types, with both progenitor and differentiated phenotypes. In the first set of experiments, we identified progenitor-like cell lines that may be involved in the phenomenon of adult pituitary plasticity, and have been able to maneuver these cells to a more mature gonadotrope cell phenotype. In the second set of experiments described, we have characterized adult gonadotrope cell lines for LHβ and α-subunit mRNA expression, LH protein expression, basal secretion of LH and gonadotropin-releasing hormone (GnRH)-mediated responses.

## Materials and Methods

### Primary pituitary cell culture

C57BL/6 mice were purchased from Charles River (Montreal, Canada). All procedures were conducted in accordance with the regulations of the Canadian Council on Animal Care and approved by the University of Toronto Animal Care Committee. Twelve week-old female littermate mice were deeply anesthetized with isoflurane. Brains were explanted and a pituitary gland was isolated and stored in fresh ice-cold HBSS dissection medium. Tissue was washed in 0.1 M phosphate buffered saline (PBS, pH 7.4), and transferred into 1 ml of growth plating media consisting of Neurobasal medium, B-27 supplement (1∶50; GIBCO Invitrogen Corporation, USA), 0.5 mM L-glutamine, 0.5% fetal bovine serum (FBS; Hyclone, USA), 25 µM glutamic acid, and 1% penicillin-streptomycin (GIBCO Invitrogen, USA), as this medium was previously shown to be effective for growth before immortalization [2], [3]. Tissues were gently triturated until uniform cellular dissociation was achieved. The suspension was plated on a 60 mm^2^ cell culture dish coated with poly-L-lysine, and placed in an incubator (5% CO_2_) for two days allowing cells to adhere.

### Immortalization and subcloning

After three days and every two days thereafter, cultures received fresh plating medium (without glutamic acid) mixed with sterile conditioned medium at a 1∶1 ratio to remove debris while cells acclimatized. Primary cultures were treated with ciliary neurotrophic factor (CNTF) for seven days, followed by transfection with recombinant murine retrovirus harboring the SV40 T-antigen and the neomycin resistance gene from the pZIPNeo SV(X) 1 vector, as previously described [2]. Retrovirus infected cells were incubated for two days followed by treatment of 400 µg/ml geneticin (G418) every 3 days for 2 weeks, then 200 µg/ml G418 for another 3 weeks. After 2 ∼ 3 weeks, G418 resistant colonies appeared and medium was changed to nutritive rich proliferating medium containing Neurobasal medium, B-27 supplement, L-glutamine, penicillin-streptomycin (as above), with the addition of 10% FBS. Upon first passage, mixed cell cultures were maintained in DMEM (GIBCO Invitrogen Corporation, USA), 10% FBS, 5% normal horse serum (heat inactivated, GIBCO Invitrogen Corporation, USA), and 1% penicillin-streptomycin. Resistant colonies were picked using cloning cylinders or serially diluted into 96-well plates and further expanded. After several passages, a mixed population of G418 resistant pituitary cells was further subcloned through successive dilutions of the trypsinized cells into 96-well tissue culture plates coated with poly-L-lysine. The optimal dilution allowed only 1 or 2 cells per well. Cells were incubated in 1∶2 conditioned medium taken from confluent mixed pituitary cell cultures. Cell colonies were allowed to grow to 90% confluence, and then successively split into 24 well plates, and finally into 60 mm dishes for RNA analysis and cryopreservation. Each cell line has been successively passaged for at least 10 generations with no change in stability or expression of pertinent markers, demonstrating a consistent, stable population of cells. Doubling time for each line was approximately 24-36 h, with minimal growth rate differences, and cells form a monolayer on tissue culture plates without the need for a matrix (such as poly-L-lysine) once established.

### Reverse transcriptase polymerase chain reaction (RT-PCR)

Each cell line was analyzed for the expression of specific markers by reverse transcriptase PCR (RT-PCR). Total RNA from each cell line was isolated by the guanidinium thiocyanate phenol chloroform extraction method, and assessed for purity and concentration using spectrophotometric analysis (Ultraspec3000, Amersham Pharmacia Biotech, USA). All RNA samples were DNase treated (Turbo DNase, Ambion Inc., Austin, TX) and then amplified using a one-step RT-PCR Kit (Qiagen, Mississauga, ON) as per manufacturer's instructions. PCR was conducted according to the following: 95°C for 30 s, 60°C for 30 s, and 72°C for 1 min (40 cycles). Annealing temperature was altered according to the corresponding primer requirements. A total of 200 ng of RNA template from each cell line was used for each reaction. All PCR-amplified products were visualized on 2% agarose gels containing ethidium bromide (final concentration of 0.05 mg/mL), under ultraviolet light. A 50 bp DNA Ladder (Fermentas Life Science, Burlington,ON) was used to determine product size. Relative gene expression levels were quantified by densitometry on images taken using an imaging station (Kodak Image Station 2000R) and analyzed using computer software (Kodak 1D Image analysis 3.6., Eastman Kodak, Rochester, NY). All primers were designed using mouse mRNA sequences and were made to cross at least one intron (Supplementary Table S1). All PCR fragments were sequenced to confirm identity.

### Cell treatments and quantitative RT-PCR

Putative progenitor cell lines mPitA-1/3, -1/6 and -3/1 were treated with either 1% or 10% serum and forskolin (100 nM, 1 uM or 10 uM) over 7 days. Treatment and medium were replaced every 2 days. Putative gonadotrope cell lines mPitA-12/3 and -12/4 were exposed to 100 nM GnRH for 6 or 24 h. Time-matched controls were included for each experiment. Total cellular RNA was isolated by the guanidium isothyiocyanate phenol choloroform extraction method. cDNA was made using the Applied Biosystems High Capacity cDNA Reverse Transcriptase Kit (Foster City, CA). Analysis of LHβ, α-subunit, FSHβ and SOX9 gene expression was completed using real-time RT PCR. Real-time RT-PCR reactions were performed with 200 ng of cDNA template using SYBR green PCR master mix and run on the Applied Biosystems Prism 7000 real-time PCR machine. LHβ primer sequences are as follows: SYBR sense 5′-tca cca cca gca tct gtg ccg-3′: and SYBR anti-sense 5′-aga tgc gaa gcg cag ctc cc-3′. FSHβ primer sequences are as follows: SYBR sense 5′-ccc agc tcg gcc caa ta-3′, and SYBR anti-sense 5′-gca atc ttc cgg tct cgt ata cca-3′. α-subunit primer sequences are as follows: SYBR sense 5′-agg cca cag taa tgg gaa atg cc-3′: and SYBR anti-sense 5′-gct cag cag ccg tca gca ca-3′. SOX9 sequences are as follows: SYBR sense, 5′-cta atg cta tct tca agg cgc tgc-3′ and SYBR antisense, 5′-acc ctg aga ttg ccc aga gtg ct-3′. Histone sequences are as follows: SYBR sense, 5′-cgc ttc cag agt gca gct att-3′ and SYBR antisense, 5′-atc ttc aaa aag gcc aac cag at-3′. Real-time RT-PCR values were normalized to histone at the corresponding time points.

For daily GnRH pulse experiments mPitA-12/3 cells were plated onto 60 mm plates at 75–80% confluence in normal growth medium. In the evening of the same day the cells were washed with 1xPBS and transferred into phenol red free medium (Hyclone, Fisher Scientific, Canada) containing 5% charcoal stripped FBS, 1% penicillin-streptomycin, 10 nM Estrogen (Sigma, Canada), 10 nM and 20 nM Dexamethasone (Sigma, Canada), or vehicle alone as a control. The following day the plates were divided into two groups, one receiving two daily 15 min pulses of 10 nM GnRH (Phoenix Pharmaceuticals, U.S.A) and the other receiving no GnRH pulses. For the cells receiving GnRH pulses the media from the previous day was replaced with phenol-red free media containing GnRH and either control or steroid treatment for 15min. Thereafter, the media was removed, the cells were washed with 1x PBS and placed in their respective treatment or control medium. This GnRH pulse procedure was repeated after a 75 min interval. The cells not receiving GnRH pulses were washed once with 1x PBS and fresh control or steroid containing media was added once daily. This protocol was conducted for three consecutive days, and on the fourth day the cells were harvested and total RNA was isolated. GnRH R mRNA levels were analyzed using semi-quantitative RT-PCR with primer sequences as follows: sense, 5′-caa tgt gtg acc cac tgc agc ttt-3′; antisense, 5′-ttt agc gtt ctc agc cga gct ctt-3′ with 60 C annealing temperature, as described above. The gel was imaged on the Kodak Image Station 2000R under UV Epi-illumination. Values for the GnRH Receptor were normalized to corresponding histone levels for each sample.

### Immunocytochemistry

Cells were seeded into 8 chamber culture slides and cultured to approximately 70% confluence, and fixed with 4% paraformaldehyde for 15 minutes. For immunocytochemical staining, cells were washed with PBS, blocked with 1% BSA and permeabilized with 0.5% Triton X-100 in PBS for 60 minutes at room temperature. The following primary rabbit- or chicken-derived polyantibodies were used: PRL (1∶200, AbD Serotec, Raleigh, NC), LH (1∶200, AbD Serotec), GH (1∶ 100, Abcam, Cambridge, MA), OXY (1∶10000, Abcam), ADH (1∶200, AbD Serotec) and ACTH (1∶200, Abcam). Slides were incubated with primary antibodies at 4°C overnight, followed by incubation with secondary antibodies for 90 minutes at room temperature. For PRL, and LH, we used FITC-conjugated goat anti-rabbit IgG (1∶100, Jackson Immuno Research, Westgrove PA). For GH and OXY, we used FITC-conjugated goat anti-chicken IgG (1∶100, Jackson ImmunoResearch). For ADH and ACTH, we used biotinylated goat anti-rabbit IgG (1∶200, Vector laboratories, Burlington, ON) and visualized with streptavidin-conjugated Texas Red. Slides were counterstained with DAPI. After washing cells with PBS, gaskets were removed from the chamber slides and mounted with DakoCytomation fluorescent mounting media. Fixed cells were then visualized and images were taken with a fluorescence microscope equipped with a CoolSNAPE CCD camera (Olympus, Japan).

### Statistical analysis

Data was analyzed by a one-way or two-way ANOVA followed by a post hoc Tukey's multiple comparison (GnRH-treatment studies) and a Student *t-test* or Mann Whitney U test (forskolin-treatment studies) using SigmaStat Software. Experiments were performed on three to four separate occasions. Data was considered statistically significant when p<0.05.

## Results

### Immortalized pituitary cell mixtures express endocrine markers

**Figure 1:**
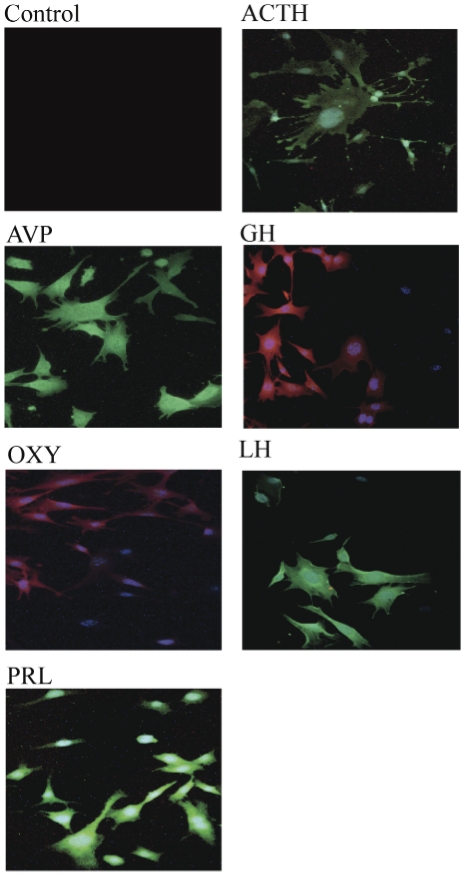
Characterization of endocrine markers in immortalized, pituitary cell mixtures. Representative immunocytochemistry images using either FITC-conjugated (shown in green) or Texas Red-conjugated (shown in red) antibodies, and counter-stained with DAPI. ACTH, adrenocorticotropic hormone; ADH, anti-diuretic hormone; GH, growth hormone; OXY, oxytocin; LH, luteinizing hormone; and PRL, prolactin.

Using a technique previously established in our lab [2], [3], we have immortalized dividing cell types present in primary pituitary culture through retroviral transfer of the SV40 T-antigen. The primary cell culture was prepared from pooled adult-derived pituitary tissue of 12 week-old female littermate mice. The immortalized mixed cells (mPitA-mix) were screened and characterized immunocytochemically using antibodies against ACTH, adrenocorticotropic hormone; ADH, anti-diuretic hormone; GH, growth hormone; OXY, oxytocin; LH, luteinizing hormone; and PRL, prolactin (**Figure 1**). These hormones were expressed in the immortalized cell mixture; however, every cell appeared to express a unique pituitary protein. For each specific hormone screened, we found positive staining in some, but not all cells that stained positive for DAPI (representative immunostained fields presented in Figure 1). This indicated a selective expression of hormones in the clusters from the mixed cell population.

### Subcloned cell lines differentially express pituitary hormones

**Figure 2:**
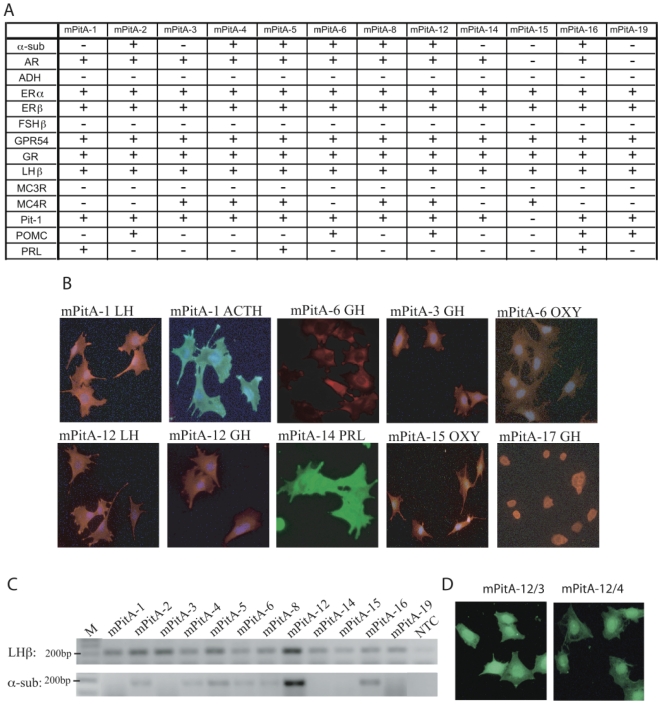
Analysis of pituitary cell markers in subcloned cell lines. (A) RT-PCR was performed on twelve of the subcloned cell lines for the indicated cellular markers. (B) Immunocytochemical analysis was performed using FITC-conjugated (shown in green) or Texas Red-conjugated (shown in red) antibodies, and counter-stained with DAPI. (C) Representative gels for LHβ (amplicon size = 196 bp) and α-subunit (amplicon size = 168 bp) are shown. M = marker; NTC = non-template control. (D) Immunocytochemical analysis using an antibody against LH was performed in the mPitA-12/3 and -12/4 cell lines.

We serially diluted and subcloned the immortalized mixed cell populations to obtain cultures containing one or more cells. We successfully subcloned 19 cell lines (mPitA-1 to 19) and characterized these lines for the expression of pituitary hormones and receptors (**Figure 2**). Using RT-PCR, we have analyzed gene expression of androgen receptor, AR; anti-diuretic hormone, ADH; estrogen-receptor β, ERβ; follicle-stimulating hormone β, FSHβ; glucocorticoid receptor, GR; luteinizing hormone β, LHβ; melanocortin-3 receptor, MC3R; melanocortin-4 receptor, MC4R; Pit-1; pro-opiomelanocortin, POMC; and prolactin, PRL (**Figure 2A**). Immunocytochemistry with antibodies against ACTH, ADH, LH, GH, OXY, and PRL was also performed in mPitA-1 to 19, (representative images shown in **Figure 2B**). Altogether, data from both RT-PCR and ICC indicate that the subcloned cell lines differentially express a variety of pituitary hormones and receptors; each line was found to express more than one pituitary hormone. This mixed expression of pituitary hormones in a specific cell line suggests a heterogeneous, non-clonal population. Our goal was to generate a clonal gonadotrope cell line; therefore we selected the mPitA-1, 3 and 12 lines based on the strong expression of LHβ, and α-subunit in the mPitA-12 line (**Figure 2C**) and further subcloned these lines in order to obtain homogeneous, clonal cell populations (**Figure 2D**).

### Forskolin, but not serum deprivation, drives differentiation of SOX2-expressing progenitor-like cell lines towards a mature endocrine cell type

**Figure 3:**
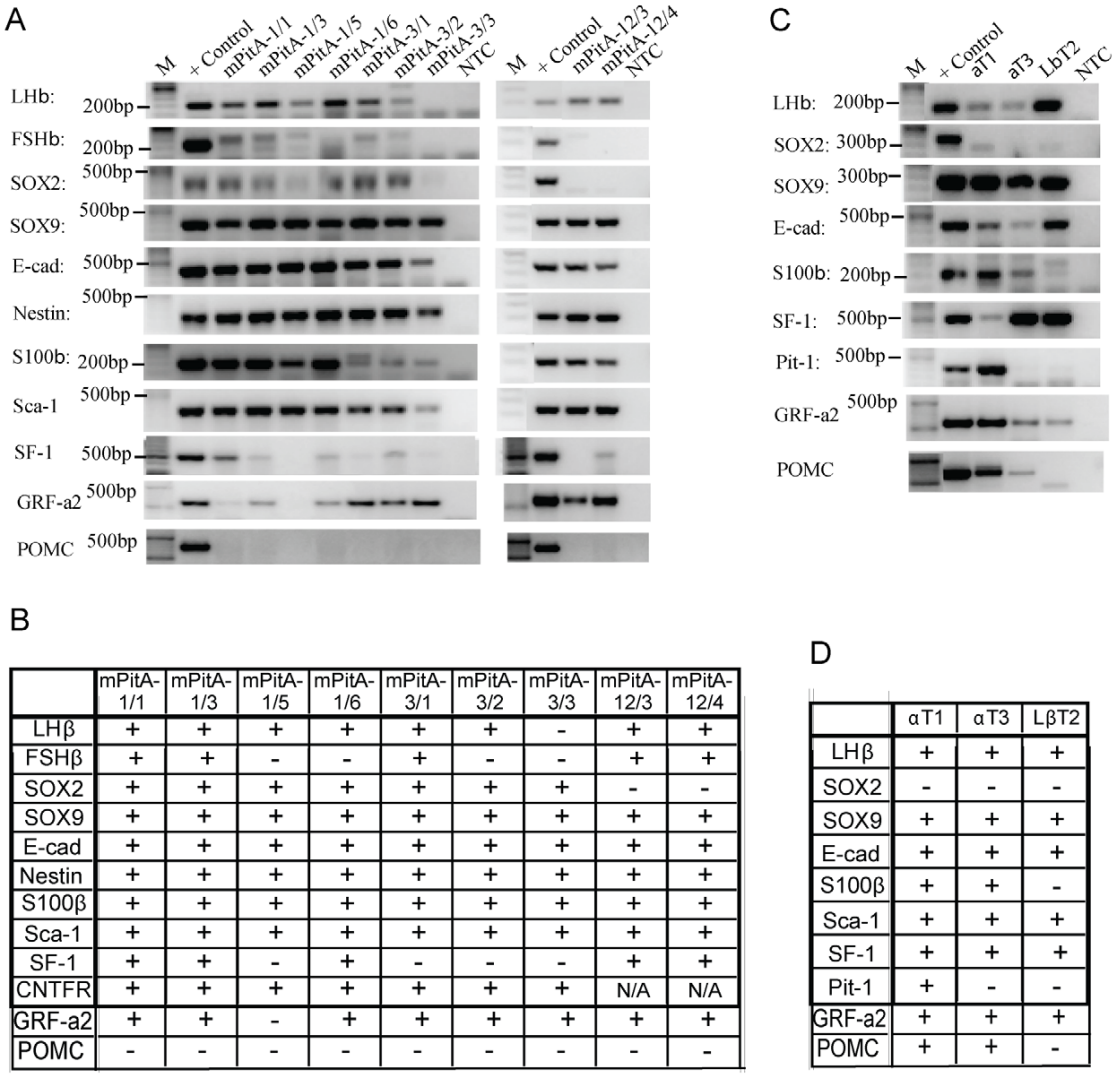
Expression of gonadotropin and progenitor cell markers in clonal cell lines. The mPitA-1, 3 and 12 cell lines were subcloned into a single cell population. (A) The cell lines were screened for gonadotropin and progenitor cell markers by RT-PCR, and results are summarized in (B). (C) Previously established progenitor or gonadotroph cell lines (αT1, αT3 and LβT2), generated by targeted tumorogenesis in transgenic mice, were screened for gonadotropin and progenitor cell markers by RT-PCR as a comparison, and results are summarized in (D).

We subcloned the LHβ- and α-subunit-expressing mPitA-1, mPitA-3 and mPitA-12 cell lines. Nine of the subclones (mPitA-1/1, 3, 5 and 6, mPitA-3/1, 2 and 3 and mPitA-12/3 and 4) were selected and further screened for the expression of LHβ and α-subunit (**Figure 3A**). In order to identify the stages of differentiation of the cell lines, we additionally screened these lines for the expression of progenitor cell markers using RT-PCR (**Figure 3A**). Subclones of the mPitA-1 and -3 lines expressed SOX2, indicating that they might consist of undifferentiated pituitary gonadotrophs [7], [8]. They also expressed other progenitor cell markers including SOX9, E-cadherin, nestin, S100β, Sca-1 and SF-1 (**Figure 3A,B**). This expression suggests that these cell lines are transit-amplifying cells (SOX2+, SOX9+, E-cadherin+, S100+) in the early stages of differentiation, as identified by Fauquier *et al.* [8]. We found expression of GRF-a2 in both putative progenitor and differentiated cell lines (**Figure 3A,B**), but found no evidence of Prop1 expression in any of the lines (data not shown), both of which have been suggested to be expressed in a cell niche of progenitor cells in the adult pituitary [17]. Importantly, the SOX2-expressing progenitor-like cell lines also expressed LHβ, but not POMC, which indicates that these lines may be undifferentiated gonadotropes. In addition to our retroviral infection-derived cell lines, we screened three previously established pituitary gonadotrope cell lines, αT1, αT3 and LβT2, for the expression of progenitor markers and did not find expression of SOX2. These cell lines did, however, express SOX9, E-cadherin, S100β, SF-1 and Pit-1 (**Figure 3C,D**). These expression patterns indicate that the mPitA-1/1, -1/3, -1/5 and -1/6 and mPitA-3/1, -3/2 and -3/3 cell lines are representative of progenitor-like cells in the pituitary.

**Figure 4:**
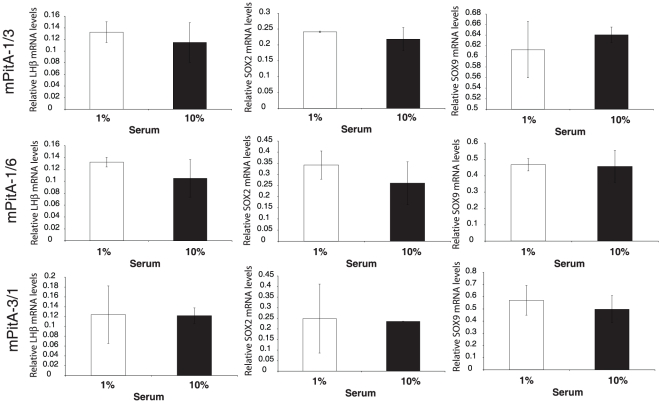
Serum deprivation does not change LHβ, SOX2 or SOX9 mRNA expression in mPitA-1/3, -1/6 or -3/1 cells. mPitA-1/3, -1/6, and -3/1 cells were exposed to 1% or 10% fetal bovine serum daily for 7 days. LHβ, SOX2 and SOX9 mRNA expression were determined using real-time RT-PCR and levels were normalized to histone. Results are expressed as mean ± SEM (n = 3-4 independent experiments).

**Figure 5:**
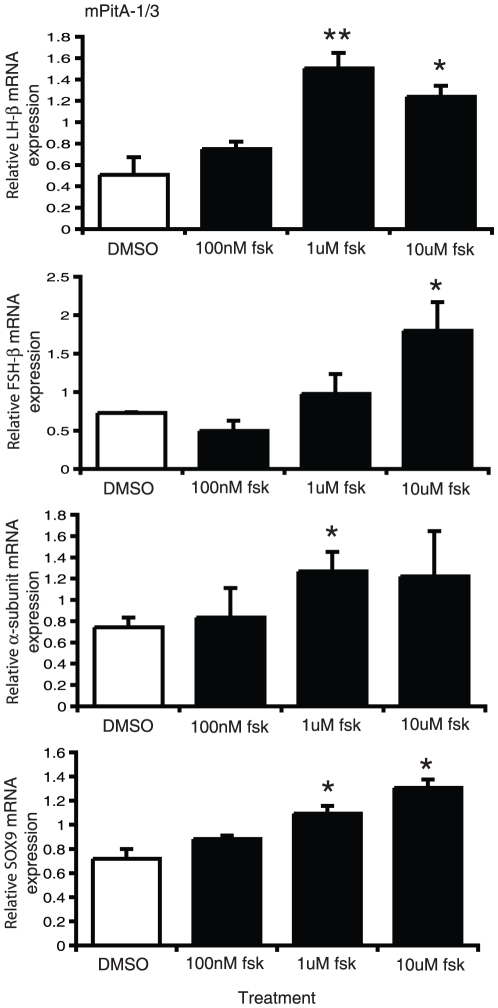
Forskolin increases LHβ, FSHβ, α-subunit and SOX9 mRNA expression in mPitA-1/3 cells. mPitA-1/3 cells were treated with forskolin (100 nM, 1 uM or 10 uM) daily for 7 days. LHβ, FSHβ, α-subunit and SOX9 mRNA expression were determined using real-time RT-PCR and levels were normalized to histone. Results are expressed as mean ± SEM (n = 3-4 independent experiments). *p<0.05, **p<0.01.

**Figure 6:**
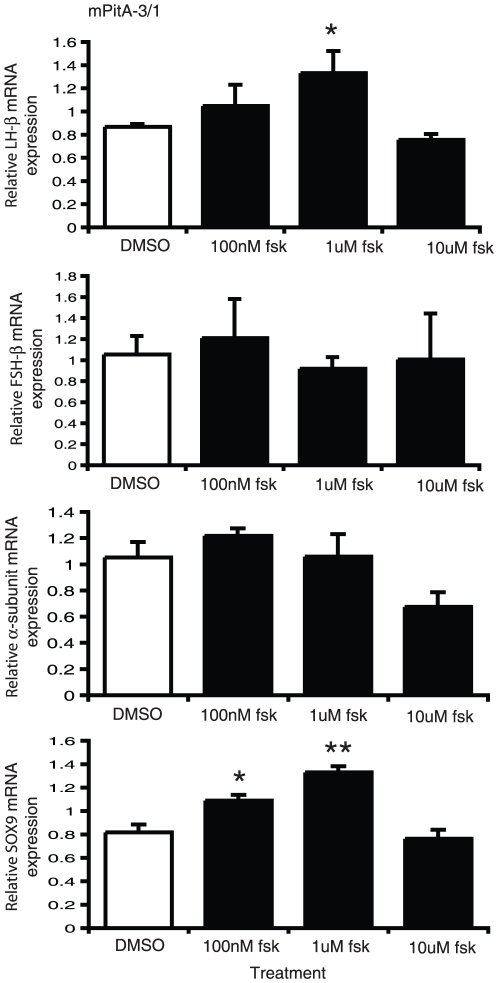
Forskolin increases LHβ and SOX9 mRNA expression in mPitA-3/1 cells. mPitA-3/1 cells were treated with forskolin (100 nM, 1 uM or 10 uM) daily for 7 days. LHβ, FSHβ, α-subunit and SOX9 mRNA expression were determined using real-time RT-PCR and levels were normalized to histone. Results are expressed as mean ± SEM (n = 3-4 independent experiments). *p<0.05, **p<0.001.

It has been postulated that serum contains components that can prevent or induce differentiation of progenitor cells; as such serum deprivation has previously been used to induce differentiation in neuronal cell cultures [18], [19], [20]. We therefore selected three of our progenitor-like cell lines based on their strong relative expression of SOX2, mPitA-1/3, -1/6 and -3/1. These cell lines were treated with either 10% (control) or 1% (serum deprived) fetal bovine serum for seven days. The medium was replaced daily. Analysis of total RNA for LHβ, SOX2 and SOX9 mRNA expression did not demonstrate any significant changes in any of the progenitor-like pituitary cell lines (**Figure 4**). Previously, the adenylate cyclase activator forskolin, through its induction of cAMP, was found to promote cellular differentiation in adult brain stem cells [21], [22], [23]. We then tested whether we could induce the differentiation potential of two of our progenitor-like cell lines, mPitA-1/3 and -3/1, with treatment of increasing concentrations of forskolin daily for seven days. Using real-time RT-PCR, we show that forskolin significantly increases LHβ, FSHβ and α-subunit expression in the mPitA-1/3 cell line, which corresponded to an increase in SOX9 expression, suggesting that these cells are undergoing differentiation (**Figure 5**). In the PitA-3/1 cell line, forskolin significantly increased LHβ and SOX9 mRNA expression, also indicating that forskolin is promoting the differentiation of these cells (**Figure 6**). These findings indicate that cell models of progenitor cells in the pituitary can be induced towards differentiation into a more mature endocrine cell type.

### Characterization of mature, clonal gonadotrope cell lines

In contrast to the putative progenitor-like cell lines, subclones of the mPitA-12 line did not express SOX2, suggesting that these cell lines, mPitA-12/3 and -12/4, are representative of mature gonadotropes (**Figure 3**). On the other hand, they did express α-subunit, LHβ, FSHβ and GnRHR (albeit FSHβ and GnRHR had low basal levels; (**Figure 3** **and** **7A**)). mPitA-12/3 and -12/4 also stained positive for LHβ using ICC (**Figure 2D**.

**Figure 7:**
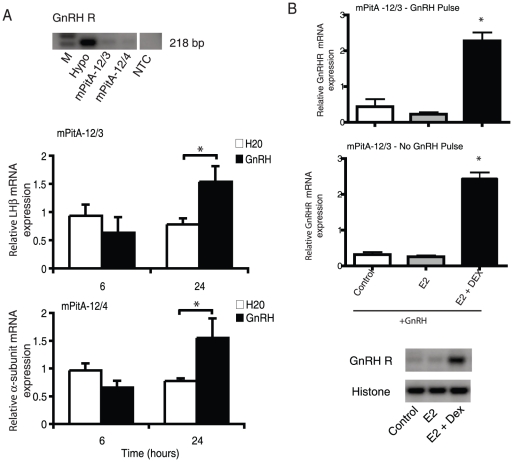
Characterization and functional analysis of gonadotrope cell lines. (A) mPitA-12/3 or mPitA-12/4 cells were screened for the presence on GnRH receptor (GnRH R) mRNA by RT-PCR. Both lines were treated with GnRH (30 nM) for 6 and 24 h. LHβ and α-subunit mRNA expression were determined using real-time RT-PCR and levels were normalized to histone. (B) mPitA-12/3 cells were treated with GnRH (10 nM) over a four-day pulse paradigm (GnRH pulse) or with a single daily treatement (no GnRH pulse), in combination with 10 nM 17β-estradiol -/+ 20 nM dexamethasone. GnRH receptor mRNA expression was determined using semi-quantitative RT-PCR and levels were normalized to histone. A representative RT-PCR gel is shown. Data are expressed as mean ± SEM, n = 3-8 independent experiments. *p<0.05.

In order to test the functionality of our gonadotrope cell lines, we tested the LHβ and α-subunit mRNA response to GnRH in the mPitA-12/3 cells. These cells were treated with 30 nM GnRH for 6 and 24 h. Total RNA was isolated at the 6 and 24 h timepoints and mRNA expression was analyzed by real-time RT-PCR. At the 24 h timepoint, LHβ and α-subunit mRNA expression were significantly increased by 2-fold compared to time-matched controls (LHβ: control 0.78±0.11 vs. GnRH 1.54±0.28; α-subunit: control 0.78±0.05 vs. GnRH 1.55±0.35) (**Figure 7A**).

When exposing the mPitA-12/3 cells to a modified pulse paradigm of 10 nM GnRH (two pulses per day for four days instead of four pulses per day for four days; [24]), in combination with 10 nM 17β-estradiol and 20 nM dexamethasone, we found that there was a significant increase in GnRH receptor mRNA levels. Upon further analysis, we found that the full pulse regimen was not necessary to increase the levels of GnRH receptor, but the addition of both 17β-estradiol and dexamethasone significantly increased the levels of GnRH receptor. The increase occurred after a daily single exposure of 10 nM GnRH to the cells over four days, and did not significantly differ from the daily two half hour pulse dose over four days; approximately 10-fold (**Figure 7B**). Thus, our adult pituitary cell lines respond appropriately to hormone stimulation and will be valuable in dissecting the molecular events involved in the neuroendocrine regulation of gonadotrope subunit synthesis.

## Discussion

The pituitary is a multifaceted organ that consists of a number of unique cell types controlling many aspects of endocrine physiology [1]. These cells represent the second order regulation of reproduction, stress, thyroid function, growth, lactation (anterior lobe) and osmotic homeostasis (posterior lobe). The anterior pituitary contains gonadotropes, corticotropes, thyrotropes, somatotropes, and lactotropes, each expressing their own unique set of hormones. The posterior pituitary secretes anti-diuretic hormone and oxytocin. The analysis of the individual cell types and their cell biology is of interest, but the available cell models are limited. For many years, a group of representative lines that express markers of specific pituitary cell lineages has been used to study aspects of pituitary function. While it is still debated whether these lines are optimal and truly representative of their in vivo counterparts, the point was often overlooked as these were the best (and only in many cases depending upon the specific cell type) cell lines available. In general, a few lines have been adopted by their fields as appropriate models, although other lines have been described (see [1] for review of all lines developed). The developmental stages of pituitary gonadotropes and their control by peripheral hormones have been studied in a few lines created using targeted SV40 T-antigen-induced tumorogenesis in mice by Mellon and colleagues using pituitary hormone gene promoters that are switched on early during cell lineage differentiation [25], [26], [27]. The α-T1 cells express only the α-subunit of the glycoprotein hormones, and appear to be the progenitor for the thyrotrope and gonadotrope lineages [25], [27], whereas the α-T3 line appears to contain more differentiated cells. However, the LβT2 and LβT4 cells have a more mature gonadotrope phenotype, as they express both α-subunit and LHβ, steroidogenic factor-1 (SF-1), as well as the GnRH-R [25], [28], and subsequent studies have shown that LβT2 cells express FSHβ [28]. Our analysis of the precursor markers expressed in these lines concurs with the developmental timelines reported previously. The reasons for apparent differences in subunit expression likely correspond to the unique origins of each of the lines. The LβT2 line was derived from a pituitary culture in a transgenic mouse, whereas our lines were derived from primary culture of adult-derived pituitary cells. Expression of T-antigen may vary since this gene is driven by different promoters as well, which could potentially change basal expression levels of certain genes. Each of these lines is also clonal and may therefore represent a unique gonadotrope cell. Thus emphasizing the importance of having more than a single cell model to study gonadotrope functionality.

As for the other cell types, there are a wide variety of lines that have been established from different species, originating from tumors or transgenically-derived, each representing a unique temporal stage of differentiation [1]. There is only one corticotrope cell line available from an ACTH-producing mouse tumor [29], [30], the AtT-20 line, which has been used extensively [1]. The thyrotrope cell lines generated from a mouse tumor, TtT-97 [31], or using SV40 T-antigen, TαT-1 [25], have not been studied as widely due to the variable expression of markers. The lactotrope cell lines include 235-1, derived from a rat tumor [32]; MMQ, also from a rat tumor [33]; whereas the GH3 cell line, derived from another rat tumor, may be considered both a lactotrope and somatotrope (somatolactotrope) due to its expression of both prolactin and growth hormone [34]. Clearly, the selection of available lines is limited and further models, preferably from adult-derived cells, are warranted. While primary culture can obviously be used as a more representative pituitary model, it is virtually impossible to study the individual cell types. Further, the cultures can be difficult to maintain, heterogeneous in nature, and the potential for variability amongst research labs is high.

In order to address this unavailability of pituitary cell models, we have generated an array of immortalized cell lines from the fully differentiated adult mouse pituitary. Immortalization of primary pituitary cell culture from a 12-week old female mouse was achieved through retroviral transfer of the SV40 T-antigen oncogene, followed by selection with geneticin. The resulting mixed cell population was serially diluted and subcloned. The subcloned cell lines were evaluated for the expression of key pituitary hormones and receptors using both immunocytochemistry and RT-PCR. Although mRNA and protein expression analysis were mostly consistent, the mPitA-1 cells stained for ACTH protein, but were not found to express POMC mRNA using RT-PCR. We have attempted to detect POMC mRNA with a number of primer sets and in many cell lines. These results can be explained by the fact that POMC mRNA is relatively unstable and undergoes post-transcriptional modifications that complicate the detection of this mRNA [35]. In addition, these cell lines are mixed cell populations that further impair the detection of POMC mRNA or it could simply be an attribute of the nature of the immortalized cell lines. These mixed lines can be further subcloned and analyzed either as clonal cell populations or as mixed cell populations. The interest in our laboratory is reproductive function, thus our specific aim was to generate a gonadotrope cell line. Therefore, we further analyzed the mPitA-12/3 cell line, which expressed LHβ mRNA, LH protein, and basally secreted LH as detected by a LH-specific radioimmunoassay. Importantly, this cell line did not express the progenitor cell marker SOX2, suggesting a fully differentiated cell type. Functional analysis of the mPitA-12/3 cell line revealed that upon treatment with 30 nM GnRH for 24 hours, LHβ and α-subunit mRNA expression was significantly increased. These results are in accordance with the well-established effect of GnRH on LH synthesis and secretion [36], [37], providing evidence that these novel cell lines are functional models to study the specific biology and control mechanisms of the differentiated pituitary gland. We used a single pulse of GnRH in the first part of our study to determine if our gonadotrope line was responsive to GnRH; however, when we varied the pulse frequency over four days, we detected a significant increase in GnRH receptor expression. This effect was synergistically enhanced by the addition of estrogen and dexamethasone, as previously shown [24], but in our case did not appear to require the increased pulse paradigm (ie. four pulses per day for four days). This is in accordance with a number of previous studies, although the reported results vary with treatment paradigm and cell types [38]. An increase in pulse frequency has been shown to enhance the overall response to GnRH [39], [40], which was not the case in our study. Unfortunately, due to the lack of an appropriate sensitive immunoassay, we were not able to follow-up the transcriptional studies with secretion analysis. Once a more sensitive assay is available commercially, these lines will be studied for regulated responses to hormones as has been shown in vivo. Until this time, we are not completely certain that the lines are mature gonadotrope models, although their origin from adult-derived pituitary and the complementary analysis of gene expression supports this theory.

An incredible database has been developed regarding pituitary organogenesis and cell differentiation. In particular, the cell-restricted expression of transcription factors involved in early pituitary development has been fairly well defined, and the expression of unique markers during the differentiation of each of the cell types from the pituitary has been mapped [41]. Gene deficiencies associated with pituitary hormone disorders have been identified [41], [42]. This impressive body of work has taken years, maybe decades, to decipher and it is not possible to summarize and do it justice in a single paragraph. Yet, there is still much to be learned, particularly with regards to the presence of stem cells, and progenitor cells, which are now thought to be present in the adult pituitary [6], [7], [8], [43]. The adult pituitary itself likely undergoes a certain amount of plasticity due to the ongoing hormonal demands during our lifespan. It has been postulated that there are progenitor cells that are committed to a specific cell lineage, and these cells can be differentiated when required by the pituitary gland [7]. For example, lactotrope numbers increase substantially during pregnancy, but the origin and mechanisms controlling the progression and differentiation of the progenitor cells is not yet understood. This is apparently the case for all pituitary cell lineages. Recent work has indicated that there may be specific cellular markers within progenitor cells, or the more committed “transit-amplifying” cells, destined for differentiation [6], [7], [8], [43]. Yet how these cells respond to specific hormones or signals that dictate their ultimate cell fate is not known; thus having models to study this process would be optimal.

Importantly then, seven of our subcloned cell lines were found to express mRNA for several progenitor cell markers. Recently, studies in both rodents and humans have identified a subpopulation of hormonally-null progenitor cells in the pituitary that can differentiate into specialized endocrine cell types (e.g. gonadotropes), allowing cellular remodeling of the pituitary in times of growth, puberty and stress in order to meet fluctuating hormonal demands [7], [8], [17], [21], [44], [45], [46], [47], [48], [49]. Although several markers for these pituitary progenitor cells have been proposed, the pluripotency transcription factor SOX2 is the strongest candidate [7]. Based on studies by Faquier *et al*. [8], multipotent progenitor cells in the pituitary are SOX2^+^/SOX9^-^, but as differentiation towards a specialized cell type occurs, as marked by the synthesis of the corresponding endocrine hormone, synthesis of SOX9 protein also increases. The resulting SOX2^+^/SOX9^+^ cells represent transit-amplifying cells that are not yet differentiated, but committed to a specific cell type. Two of our cell lines, mPitA-1/3 and 3/1 may be transit-amplifying cells committed to the gonadotroph cell type. Our results did not agree completely with studies by Garcia-Lavandeira et al. where they found that a population of putative pituitary stem/progenitor cells distinctly expresses both GRF-a2 and Prop1 [17]. In our study, we found expression of GRF-a2 in both putative progenitor and differentiated cell lines, but found no evidence of Prop1 expression in any of the lines, which may ultimately correspond to the specific stage of development in which the cells were studied, for instance stem versus transit-amplifying cells, and perhaps the specific location/origin of the cells studied. Further, our study captured the entire adult pituitary in culture, and not a single cell niche. Further studies will be required to sort out these differences, however, we continued the study of our putative progenitor cells based on progress to differentiation by treatment with forskolin, an adenylate cyclase activator, as previously demonstrated by Weiss *et al.* [21]. After 7 days of daily treatment with forskolin, LHβ, FSHβ, α-subunit and SOX9 mRNA expression were significantly elevated in the mPitA-1/3 cell line, and LHβ and SOX9 was increased in the mPitA-3/1 cell line. These findings are in accordance with other recent studies investigating multipotent adult progenitor cells in the pituitary [8], and will be useful for further investigation into the cellular mechanisms involved in pituitary remodeling. An increase in cAMP can be achieved by a number of G protein-coupled receptors; so an obvious candidate for this process is pituitary adenylate cyclase -activating peptide (PACAP), as it is known to increase cAMP through adenylate cyclase and also induce neurogenesis [12], [13]. Yet, a 7-day exposure to PACAP, in a similar protocol to that used for forskolin, did not induce differentiation in our progenitor cell lines (data not shown). Thus, further work will be done to understand the upstream signals that drive the transit-amplifying cells towards the more differentiated gonadotrope.

We have therefore used our retroviral transfer technology to generate unique cell models from adult-derived pituitary primary cell culture. Since these cells were not the result of a pituitary tumor (either natural, radiation-induced, or SV40 T-antigen-induced), we expect that the cell biology should be more similar to that of their in vivo counterparts. We have been able to characterize many of our mixed cell populations and clonal cell lines, but the mixed cell lines can be further subcloned to provide clonal models of a number of the pituitary cell lineages. While there is obviously still much to be done with each of these models, we have been able to work with specific cell lines, that of the progenitor cells and of the more differentiated gonadotrope cell, to elucidate potential mechanisms by which these cells can be driven towards a specific cell fate and functionality. The dynamic function of each pituitary cell and their role in overall physiology represents a complex interplay between hormones and cellular secretion. We suggest that the use of these cell models will provide an experimental framework to study the mechanisms involved in these interactions and how the pituitary maintains its plasticity during times of growth, stress, and reproductive function. In our opinion, this new availability of immortalized cell lines from the adult pituitary will be pivotal in understanding the complex cellular mechanisms underlying progenitor differentiation, as well as cellular control of hormone synthesis in differentiated cell lines.

## Supporting Information

Table S1 **Primer sequences used for RT-PCR screening.** All RNA samples were DNase treated and then amplified using a one-step RT-PCR Kit as per manufacturer's instructions. PCR was conducted according to the following: 95°C for 30 s, 60°C for 30s, and 72°C for 1min (40 cycles). Annealing temperature was altered according to the corresponding primer requirements. A total of 200 ng of RNA template from each cell line was used for each reaction. All PCR-amplified products were visualized on 2% agarose gels containing ethidium bromide (final concentration of 0.05 mg/mL), under ultraviolet light. All primers were designed using mouse mRNA sequences and were made to cross at least one intron. All PCR fragments were sequenced to confirm identity.
(PDF)
