# Supplementary material for: Generation of Immortal Cell Lines from the Adult Pituitary: Role of cAMP on Differentiation of SOX2-Expressing Progenitor Cells to Mature Gonadotropes
Source: PLoS One. 2011 Nov 21;6(11):e27799. doi: 10.1371/journal.pone.0027799 (PMC3221660; doi:10.1371/journal.pone.0027799)
Supplement: Table S1 — Primer sequences used for RT-PCR screening. All RNA samples were DNase treated and then amplified using a one-step RT-PCR Kit as per manufacturer's instructions. PCR was conducted according to the following: 95°C for 30 s, 60°C for 30s, and 72°C for 1min (40 cycles). Annealing temperature was altered according to the corresponding primer requirements. A total of 200 ng of RNA template from each cell line was used for each reaction. All PCR-amplified products were visualized on 2% agarose gels containing ethidium bromide (final concentration of 0.05 mg/mL), under ultraviolet light. All primers were designed using mouse mRNA sequences and were made to cross at least one intron. All PCR fragments were sequenced to confirm identity. (PDF) [file pone.0027799.s001.pdf]

Supplementary Table 1 - Primer sequences used for RT-PCR screening.

Gene	Primer Sequence	Amplicon Size (bp)	Annealing Temp (∞C)	Cross Intron
ADH	S: TGC TAC TTC CAG AAC TGC CCA AGA<br>AS: AGCTGTACCAGCCTTAGCAGCA	389	60	no
a-subunit	S: ATG CAG CTG TCA TTC TGG TCA TGC<br>AS: TGG AGA AGC AAC AGC CCA TAC ACT	168	60	yes
AR	S: ACG TCC TGG AAG CCA TTG AGC C<br>AS: CTT GGT GAG CTG GTA GAA GCG C	560	60	yes
CNTF-R	S: ATA CTG CGA AGC TTA GAA CTG GGC<br>AS: GCA CAG TCA CAT TGA AGG TAT TGG	214	57	yes
E-cad	S: TGA CAA CAG GCC AGA GTT TAC CCA<br>AS: TAC GTG CTT GGG TTG AAG ACA GGA	372	60	no
ERa	S: GAA TTC AAT TCT GAC AAT CGA CGC CA<br>AS: GAA TTC GTG CTT CAA CAT TCT CCC TC	344	60	yes
ERb	S: CAG TAA CAA GGG CAT GGA AC<br>AS: GTA CAT GTC CCA CTT CTG AC	243	60	no
FSHb	S: GAA GGA AGA GTG CCG TTT CTG CAT<br>AS: TGC CAC AGT GAC ATT CAG TGG CTA	218	60	no
GPR54	S: TGG TTC CCC TGT TTT TCG CTA<br>AS: CAG CGG GAA CAC AGT CAC ATA	314	60	yes
GR	S: TGG ATA TTC AAG CCC TGG AA<br>AS: AAG ACA TTT TCG ATA GCG GC	271	60	yes
LHb	S: TGT CCT AGC ATG GTC CGA GT<br>AS: GAC CCC CAC AGT CAG AGC TA	196	60	yes
MC3R	S: TGA ACT CTT CCT GCT GCC TGT CTT<br>AS: ACC GAA GGG CAT AGA AGA TGG TGA	453	60	no
MC4R	S: ACG CGC TCC AGT ACC ATA ACA TCA<br>AS: ACG CAG TAT GGA TTC TGA GGG CAA	376	60	no
Nestin	S: AAA GTT CCA GCT GGC TGT GG<br>AS: AAA GCC AAG AGA AGC CTG GGA A	205	58	yes
Pit-1	S: AGT TTA AGC AGG AAC TCA GGC GGA<br>AS: TCC TCT TCC TTT CGT TTG CTC CCA	330	60	yes
POMC	S: TAG ATG TGT GGA GCT GGT GC<br>AS: CAG TCA GGG GCT GTT CAT CT	149	60	yes
PRL	S: TGT TCA GCC TCT GCC AAT CTG TTC<br>AS: AAG AAC TTC CGG AGG GAC TTT CAG	244	60	yes
S100b	S: CCA TGG TTG CCC TCA TTG ATG TCT<br>AS: CCA TGA ACT CCT GGA AGT CAC ACT	207	60	no
Sca-1	S: AAA GAG CTC AGG GAC TGG AGT GTT<br>AS: TAC ATT GCA GAG GTC TTC CTG GCA	254	60	no
SF-1	S: CAA GCG CAC AGT CCA GAA CAA CAA<br>AS: TTG ATG GTG CGG TTA GAG AAG GCA	471	60	no
SOX2	S: AAC ATG ATG GAG ACG GAG CTG AAG<br>AS: TCC GGG AAG CGT GTA CTT ATC CTT	387	60	no
SOX9	S: CAG CAA GAA CAA GCC ACA CGT CAA<br>AS: TTG TCC GTT CTT CAC CGA CTT CCT	265	60	no
